# Long non-coding RNAs learn the importance of being *in vivo*

**DOI:** 10.3389/fgene.2014.00045

**Published:** 2014-03-04

**Authors:** Jhumku D. Kohtz

**Affiliations:** Developmental Biology and Department of Pediatrics, Lurie Children's Research Center, Feinberg School of Medicine, Northwestern UniversityChicago, IL, USA

**Keywords:** Evf2, lncRNA knockout mice, neural development, transcription, lncRNA antisense regulation, pain modulation, microbial susceptibility, lung cancer

In the past few years, the long non-coding RNA (lncRNA) field has been dealt some major surprises. While some phenotypes of mice lacking lncRNAs reveal potential targets for treating diverse human diseases, others do not match the expectations from experimental manipulations in cell lines reported over the last 10 years. In effect, it has become clear that principles learned about lncRNA functions in cell lines can be very different when tested in animal models (*in vivo)*.

The imprinting/dosage compensation and developmental biology fields, older and wiser crowds, are rolling their eyes.

Historically, there were a small number of well-characterized lncRNAs. Among these were the classic lncRNAs *Xist*, *roX, H19, Air, KCNQ1OT1*, and *UBE3a* (Lee and Bartolomei, 2013). These lncRNAs regulate imprinting and/or dosage compensation, and were studied almost exclusively in animal models (mice or Drosophila). In 2006, elegant studies in Drosophila showed that *trans*-acting lncRNAs (TRE's) regulate transcription of the Ubx1 homeodomain transcription factor (Sanchez-Elsner et al., 2006). Along with the explosion of lncRNAs identified in the genomic era, *trans*-acting transcriptional activities of vertebrate lncRNAs, *SRA* (Lanz et al., 1999), *Evf2* (Feng et al., 2006), and *HOTAIR* (Rinn et al., 2007) were reported. However, unlike the majority of previous lncRNA experiments, initial *SRA*, *Evf2* and *HOTAIR* studies relied on cell lines to assay for lncRNA activity. “*Trans*” activities gave these lncRNAs the potential for global effects, distinguishing them from their *cis*-acting imprinting/dosage compensating counterparts.

In 2004, the *Nature* editor refused to send our paper on *Evf2* lncRNA *trans*-acting transcriptional activity out for peer review, stating that a knockout mouse model was necessary. This was not unexpected, as “knockout first, ask questions later,” had been the *modus operandi* at the NYU Skirball Institute, where scientists (including myself) were indoctrinated regarding the importance of *in vivo* studies. Thankfully, Terry Grodzicker, the editor at *Genes and Development* did not share the *Nature* editor's views, and agreed to send our paper out for peer review. This led to publication of our work on *Evf2 trans*-acting activity in 2006 (Feng et al., 2006).

In retrospect, views at Skirball and *Nature* may have been correct: *Evf2* cell line assays predicted lncRNA enhancer activation in *trans* (Feng et al., 2006), while *Evf2*^TS/TS^ mice (lacking *Evf2*) a few years later indicated lncRNA repression in *cis* (Bond et al., 2009). In mice, *Evf2* recruits both transcriptional activator (DLX's) and repressor (MECP2), and through anti-sense regulation represses adjacent gene expression (Bond et al., 2009). Recent experiments show that *Evf2* prevents enhancer CpG site-specific methylation, in *trans*, but that methylation effects may not be sufficient to regulate gene expression (Berghoff et al., 2013). Both loss-of-function and gain-of-function *Evf2* mouse models, as well as additional mouse models lacking Dlx1/2 and Mecp2, support the proposed mechanism (Berghoff et al., 2013). Relevant to ongoing studies, mice lacking *Evf2* have reduced inhibition in the adult brain, resulting from developmentally generated interneuron defects (Bond et al., 2009). Taken together, *Evf2* work suggests that lncRNA-dependent positive and negative transcription factor recruitment and enhancer DNA methylation inhibition contribute to gene dosage regulation, rather than essential gene regulation (Mattick, 2013). Mice lacking *Evf2* exhibit a different adult phenotype than would have been predicted from studies in cell lines. While demonstrating *Evf2* activity in cell lines was critical in prompting and designing subsequent work, present models for the role of *Evf2* in transcription and neuronal development rely on results obtained in mice.

Mice lacking the well-characterized lncRNAs, *NEAT1*, required for paraspeckles (Nakagawa et al., 2011), *MALAT1*, localized in nuclear speckles, (Nakagawa et al., 2012), or *HOTAIR*, recruitment of histone modification complexes that regulate Hox genes (Schorderet and Duboule, 2011; Li et al., 2013), also challenge previous data obtained in cell lines.

Loss of *NEAT1* in mice shows that paraspeckles, previously thought to be a critical subnuclear compartment, are not necessary for mouse development (Nakagawa et al., 2011). Mice lacking *MALAT1* (*NEAT2*), previously thought to be critical for nuclear speckles and splicing, show no morphological alterations (Nakagawa et al., 2012). However, a dramatic phenotype is reported in *MALAT1* conditional knockout (cKO) mice and in mice treated with anti-sense oligos to *MALAT1* (Eissmann et al., 2012). Both methods to reduce *MALAT1* substantially reduce lung tumor metastasis (Eissmann et al., 2012). In the MALAT1cKO model, gene expression adjacent to *MALAT1* is affected, but not global splicing (Eissmann et al., 2012). Since MALAT1cKO mice remove a piece of DNA in addition to removing the MALAT1 transcript, *cis*-gene effects resulting from DNA loss cannot be distinguished from RNA loss. The latter effect will need to be tested in the Nakagawa MALAT1 mice where a triple polyA (Transcription Stop, TS, Soriano, 1999) insertion prevents lncRNA expression.

There is a similar problem with *HOTAIR* loss-of-function mouse models (Schorderet and Duboule, 2011; Li et al., 2013), as well as a recent screen for novel lncRNAs (Sauvageau et al., 2013), where DNA deletion rather than TS insertion is utilized. In HOTAIRcKO mice, removal of both HoxC and *HOTAIR* does not change HoxD H3K27me3 profile or gene expression in E13.5 embryos (Schorderet and Duboule, 2011). HOTAIRcKO skeletal phenotypes and gene regulatory phenotypes are mild, with 2-fold or less changes in HoxD10 and HoxD11 expression (Li et al., 2013). However, when *HOTAIR*^−/−^ cells are placed in culture, significant differences in HoxD gene expression and H3K27me3 profile are detected, suggesting different roles of *HOTAIR* in cell lines and *in vivo* (Li et al., 2013).

Given that so many of the recent lncRNA models use cKO's to remove lncRNA from mice, an important point to address here is how lncRNA biologists choose to remove lncRNA expression from mice. cKO mice using cre-directed removal have the advantage of tissue—and developmental—stage-specific loss, avoiding prenatal and heterozygote lethality. However, in the absence of rescue, determining whether phenotypic effects result from RNA or DNA loss is not possible. If an lncRNA works in *cis*, rescue is unlikely to change gene expression. One example is our transgenic rescue experiments, where *Evf2* expressed from a transgene in mice lacking endogenous *Evf2* (*Evf2*^TS/TS^) rescues enhancer methylation, but not *cis* gene expression effects (Berghoff et al., 2013).

In addition to avoiding DNA removal, TS insertion is an efficient means of terminating lncRNA transcription, as first reported for *Tsix* (96% *Tsix* RNA reduction) (Luikenhuis et al., 2001); TS insertion was also used to terminate *AIR* expression in mice and determine the role of *AIR* in imprinting in mice (Sleutels et al., 2002). A number of lncRNA models, including *Evf2*^TS/TS^ (Bond et al., 2009) have successfully used TS to terminate lncRNA expression. Therefore, unless embryonic lethality of heterozygotes is predicted, TS insertion is the method of choice for preventing lncRNA transcription in mice.

Two very different and exciting reports of lncRNA *in vivo* significance were recently published (*NeST* Gomez et al., 2013 and *Kcna2AS* Zhao et al., 2013). In the first report, *NeST*, an lncRNA encoded by the murine viral susceptibility locus, *Tmevp3*, controls Salmonella susceptibility and alters interferon-γ H3K4me3 (Gomez et al., 2013). *NeST* was identified based on differences in microbial susceptibility between two congenic strains of mice (B10.S and SJL/J), and demonstrates the power of genetics and lncRNA biology when combined (Gomez et al., 2013). The *Kcna2AS* is an antisense lncRNA that negatively regulates Kcna2, a voltage-dependent potassium channel expressed in afferent neurons (Zhao et al., 2013). Knockdown of *Kcna2AS* reduces neuropathic pain in a rat model, identifying a novel target for pain modulation (Zhao et al., 2013). Results from *NeST*, *Kcna2AS*, and *MALAT1* lncRNAs have major implications in developing treatments for infectious, and neurological disease, as well as lung cancer.

While the arguments for utilizing mouse models to study lncRNA mechanism and significance are clear, there are several arguments, in addition to the discovery argument, to continue studies in cell lines. For instance, in the field of regenerative medicine, lncRNAs have the potential to guide human or mouse embryonic stem cells toward specific lineages, or reprogram induced pluripotent stem cells. Work on lncRNAs controlling retinal fate specification in mice *RNCR2 and Six3OS* (Rapicavoli et al., 2010, 2011), predicted that lncRNAs may be used to guide embryonic stem cell differentiation, *in vitro*. Although its role in vivo has yet to be determined, the Braveheart (*Bvht*) lncRNA directs cardiovascular lineage commitment in embryonic stem cells (Klattenhoff et al., 2013), a holy grail in the cardiac field. While studies of human—or primate-specific lncRNAs may not yield useful information in rodent models, manipulation in human embryonic stem cells may reveal their functions. The cross-information obtained from *in vitro* and *in vivo* studies are likely to be most powerful when generated in the right system for the right purpose.

## Conclusions

Although one may dispel the differences between lncRNA activities in cell lines and *in vivo* described above as a biological anomaly, such differences are not specific to lncRNA studies. The REST conditional mouse knockout serves as a salient example of how a whole field can be surprised and challenged when a key *in vivo* experiment refutes previous dogma (Aoki et al., 2012). Going against a long-standing belief that REST plays a critical role in neurogenesis, Aoki et al. (2012) show that REST is only required to repress neuronal genes in non-neuronal cells, but not in neuronal progenitors, *in vi*vo.

Determining biological significance using *in vivo* models is not only important to grant reviewers, NIH program officers, the editors of some journals, and human disease, but it is important for answering questions that eventually establish the basic principles in the field. In the case of modern lncRNAs, mechanistic studies in cell lines have so far outweighed studies in mice. However, multiple *in vivo* models are shaking up some of the previous lncRNA dogma, revealing lncRNA biological significance and functional diversity, as well as guiding the future of the lncRNA field.
